# Preparation of Immunotherapy Liposomal-Loaded Thermal-Responsive Hydrogel Carrier in the Local Treatment of Breast Cancer

**DOI:** 10.3390/polym11101592

**Published:** 2019-09-29

**Authors:** Hsieh-Chih Tsai, Hsiao-Ying Chou, Shun-Hao Chuang, Juin-Yih Lai, Yi-Shu Chen, Yu-Han Wen, Lu-Yi Yu, Chun-Liang Lo

**Affiliations:** 1 Graduate Institute of Applied Science and Technology, National Taiwan University of Science and Technology, Taipei 10607, Taiwan; wherelove8@gmail.com (H.-Y.C.); smallwhite90301@gmail.com (S.-H.C.); jylai@cycu.edu.tw (J.-Y.L.); 2 Advanced Membrane Materials Center, National Taiwan University of Science and Technology, Taipei 10607, Taiwan; 3 R&D Center for Membrane Technology, Chung Yuan Christian University, Taoyuan 32023, Taiwan; 4 Department of Biomedical Engineering, National Yang-Ming University, Taipei 112, Taiwan; kerry840726@gmail.com (Y.-S.C.); qooaz0815@hotmail.com (Y.-H.W.); smallfish410048@hotmail.com (L.-Y.Y.)

**Keywords:** liposomes, immunotherapy, temperature-sensitive hydrogel, injectable

## Abstract

To reduce the side effects of immune drugs and the sustainable release of immune drugs on local parts, we have designed an injectable thermal-sensitive hydrogel containing an imiquimod-loaded liposome system. In the extracellular environment of tumor tissues (pH 6.4), 50% of the drug was released from the carrier, which could be a result of the morphological changes of the liposomal microstructure in the acidic environment. According to the results in animals, the drug-containing liposomes combined with hydrogel can be effectively applied in breast cancer therapy to delay the growth of tumors as well as to dramatically reduce the death rate of mice.

## 1. Introduction

In 1893, it was proposed that the immune system could detect tumors and control their growth [1]. Since then, cancer immunotherapy has become a hot research field in cancer treatment. In recent years, patients with terminal cancers who underwent therapies that produced no effect were subjected to clinical immunotherapy. It was found that immunotherapy could indeed improve the survival rate of cancer patients. At present, imiquimod (IMQ) is used clinically to treat basal cell carcinoma. IMQ is an agonist of toll-like receptors (TLRs) and a derivative of the synthesized molecule, imidazoquinoline. In immunotherapy, IMQ can stimulate TLR7 and TLR8 on the surface of dendritic cells to make the cells express a high amount of cytokine and type I interferon, which then activate T cells and natural killer cells to perform immunotherapy [1]. In addition, macrophages can be activated by IMQ and become M1 macrophages [2]. While tumor tissues utilize M2 macrophages to repair tissues and facilitate growth, M1 macrophages can excrete TNF-α and a series of interleukins that lead to the death of cancer cells [3]. IMQ is an effective breast cancer immunotherapy drug that can be used in combination with radiation therapy to achieve a synergistic therapeutic effect. In the meantime, the low cost of IMQ may alleviate the burden of the high healthcare cost of immunotherapy. For patients with cancer, the systemic toxicity brought on by immunotherapeutic drugs is as serious as that of the chemotherapeutic drugs. It is thus very important to understand how to avoid the systemic damage caused by immunotherapeutic drugs and make them effective anticancer drugs through hydrogel topical delivery. 

A hydrogel is constructed by a 3D network and soft matter with extremely high water content as well as a similar structure to human tissue. However, the properties of hydrogels are based on the regulation of physical and chemical crosslinks as desired for the development of smart hydrogels [4]. One approach to addressing this challenge is an in situ localized delivery system, loading the therapeutic agent in an injectable and biodegradable hydrogel. Though various polymer-based drug carriers have been used to promote the delivery of the drug to target tissues, the local treatment with hydrogel has significantly numerous advantages over the systemic administration and polymeric drug delivery. Local drug delivery based on the injectable hydrogels could control the delivery of sustained release as well as provide high drug concentration at the target site and minimal nonspecific distribution of drugs in normal organs [5,6,7]. 

Among polymer-based carriers (micelles and liposomes), the liposome carrier features a relatively higher drug-loading capacity which may resolve the issue of poor drug delivery inside of the human body and increase the amount of drug accumulated in the tumor microenvironment. Liposome carriers can be incorporated with acidic, basic, or temperature-responsive functional groups [8,9], allowing the carried drug to be released in a specific environment, such as the mildly acidic environment (pH 6.5) of tumor tissues. To achieve a sustained and slow release of the drug carrier in the tumor microenvironment, in this study, the IMQ drugs were first incorporated in 1,2-dipalmitoyl-sn-glycero-3-phosphatidylcholine (DPPC) phospholipid liposomes, and after that, the drug carrier was incorporated in an amphipathic temperature-sensitive hydrogel, pluronic F127 (PF127). When the concentration of the PF127 aqueous solution is above the critical micelle concentration, this copolymer forms polymer micelles which can be used to embed drugs or contrast agents [10,11]. Once the concentration exceeds the gel-forming concentration, the hydrogel would be formed for topical drug delivery application [12]. Both micelles and hydrogel are formed in a temperature-sensitive manner. At low temperatures, the polymer chain interacts with water molecules through hydrogen bonds and is soluble, whereas at high temperatures, hydrophobic segments gather to form micelles and a hydrogel [13,14,15,16]. Therefore, by utilizing the high drug-loading capacity and the long-term and slow release properties of PF127, we hoped to achieve a sustained and slow release of immunotherapeutic drugs in the breast cancer tumor region and thus suppress the tumor’s growth. 

## 2. Materials and Methods

### 2.1. Materials

1,2-Dipalmitoyl-sn-glycero-3-phosphatidylcholine was purchased from Avanti® Polar Lipids, INC in Alabama, USA. Imiquimod (IMQ) was purchased from Alfa Aesar in Lancashire, England. Pluronic® F-127 was purchased from Sigma-Aldrich. Phosphate buffer solution (0.01M) (PBS) was prepared from the mixing of 137mM NaCl, 2.7 mM KCl, 10 mM Na_2_SO_4_, and 2 mM KH_2_PO_4_ in the 800 mL distilled water and then tune the pH to 7.4 with HCl solution. All the salts used here were purchased from Sigma-Aldrich. 

### 2.2. Liposome Preparation

1,2-Dipalmitoyl-sn-glycero-3-phosphatidylcholine and IMQ were dissolved in a methanol/dichloromethane solution, and the organic solvent was removed on a rotary evaporator to prepare the film. Phosphate buffer solution (PBS) was then added to perform hydration on a sonicator to form liposomes. Finally, the product was passed through a 0.22 μm membrane filter to obtain drug-containing liposomes. Unembedded drugs were removed by centrifugation (6000 rpm, 10 min) using a 3 kDa centrifugal concentrator.

### 2.3. Stability Analysis

The liposome solution was transferred into cuvettes after adjusting its pH to 7.4 and 6.5. Then the cuvettes were placed in a 37 °C microprogrammable shaking incubator under slow shaking. The particle size and size distribution were analyzed by dynamic light scattering at 0, 1, 3, 6, 12, 24, and 48 h, and the variation was calculated by dividing the particle diameter measured at different time points by that measured at 0 h. In addition, a small quantity of the sample was fixed onto a copper grid, stained with uranyl acetate, and analyzed by TEM for its morphology.

### 2.4. Exploration of Drug Release

The liposome solution was transferred into dialysis bags (molecule weight cut-off 6000~8000 Da) and then into sample bottles with PBS at different pH values. The sample bottles were then shaken at a constant speed in a 37 °C microprogrammable shaking incubator with the PBS solutions being replaced at 1, 3, 6, 12, 24, and 48 h. The solutions taken out were freeze-dried, dissolved in the methanol/dichloromethane solution, and analyzed in an ultraviolet/visible spectrophotometer at a wavelength of 246 nm.

### 2.5. Cell Test

Mouse breast cancer cells 4T1 were distributed in 96-well plates at a density of 2 × 10^4^ cells/mL. After about 8 h, when the 4T1 cells attached, the culture medium was replaced with fresh medium containing drug carriers, and the (3-(4,5-Dimethylthiazol-2-yl)-2,5-diphenyltetrazolium bromide (MTT) colorimetric assay was performed after 24 h.

### 2.6. Sol–Gel Transition Phase Diagram of PF127 Hydrogel

Different concentrations of PF127 aqueous solutions were prepared and placed in a 10 °C chilled water tank and the gel formation situation was recorded at every 2 °C increment and plotted in the diagram.

### 2.7. Rheological Measurements of Drug-Containing Liposomes Mixed with PF127

A rheometer was used to check the gel formation temperature of the drug-containing liposome–PF127 mixture. Due to its temperature-sensitive feature, the viscosity of PF127 may change with temperature. In our study, the solution was heated in a rheometer from 20 °C to 50 °C, and the viscosity changes were measured with the liquid being spun at a frequency of 0.1 (1/ms).

### 2.8. Animal Test

The mouse breast cancer 4T1 cell culture was adjusted to a density of 2 × 10^7^ cells/mL and injected in the subcutaneous position of the mouse’s second mammary gland using a 27G needle. The tumor growth situation was monitored every two days. After the tumor reached 1 cm^3^, the mice were divided into four groups, which were: (1) control, (2) in situ injection with hydrogel, (3) in situ injection with hydrogel combined with liposomes, and (4) intravenous injection with liposomes. The dosing concentration of IMQ was 0.83 mg/kg. After dosing, the tumor size and weight changes of each group were recorded every two to three days, and the test mice were sacrificed to obtain tumor tissues for analysis at the end of the test.

## 3. Results and Discussion

With respect to the nanoparticle carrier preparation, in this study, we successively applied thin-film preparation, hydration, and filtration techniques to prepare IMQ-containing liposomes. The basic properties of the prepared IMQ-containing liposomes were characterized using a particle-size analyzer. The particle size of IMQ-containing liposomes was approximately 96 nm, and the size distribution was 0.25. Although the acquired liposomes had a relatively wide size distribution, the particle size and distribution were still acceptable as the carrier did not accumulate at the tumor directly through blood circulation but had to be injected in situ in the tumor area in combination with hydrogel. On the other hand, the zeta potential of the liposomes was −6.25 mV as determined by a zeta potential analyzer. As a result, these liposomes could directly act on cancer cells after dissociation of hydrogel in the topical tumor environment. Furthermore, the drug content was identified to be 3.9 wt %.

**Figure 1:**
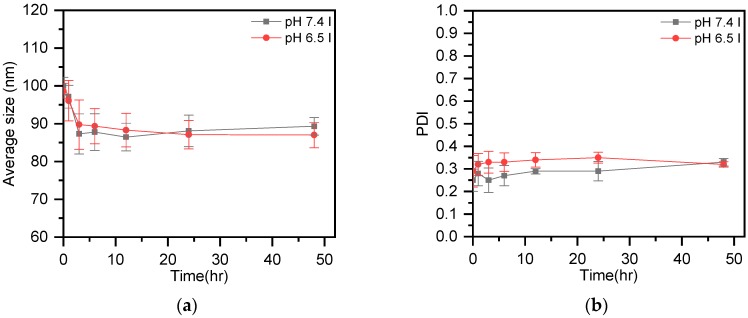
The particle size distribution variation of IMQ-containing liposomes in different pH environments.

**Figure 2:**
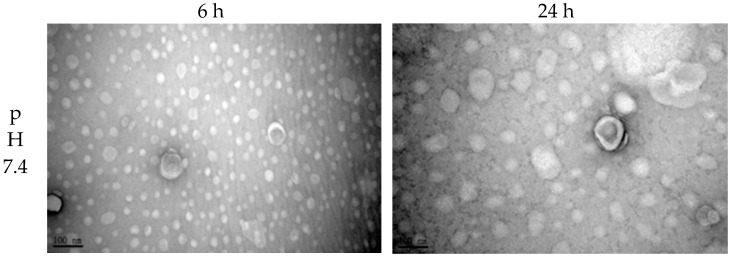

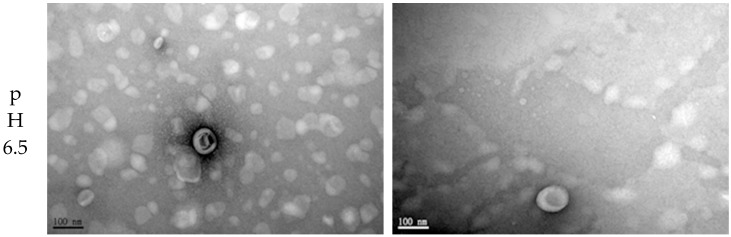
TEM images of IMQ-containing liposomes in different pH environments.

In the next step, in order to understand liposome stability in the neutral environment of the human body and the acidic surroundings of the tumor microenvironment, this study tested the variation of particle size and size distribution of liposomes at pH 7.4 and pH 6.5. According to the results shown in Figure 1, the particle size and size distribution of IMQ-containing liposomes did not undergo severe changes in either a neutral pH environment or a mildly acidic environment like that of tumors. Observations at Day 2 revealed that the particle size varied by 15% in the above-mentioned pH environments (from 100 nm to 85 nm), whereas the particle size distributions varied from 10% to 30%. The limited variations observed for particle size and size distribution were due to the weak negative charges on the nano-sized drug surface, which prevented liposomes from clustering and fusing. Further, according to the TEM images shown in Figure 2, whether at 6 or 24 h, the liposomes maintained their lipid bilayer status in both pH 7.4 and pH 6.5 environments, and the particle size did not undergo great changes.

**Figure 3:**
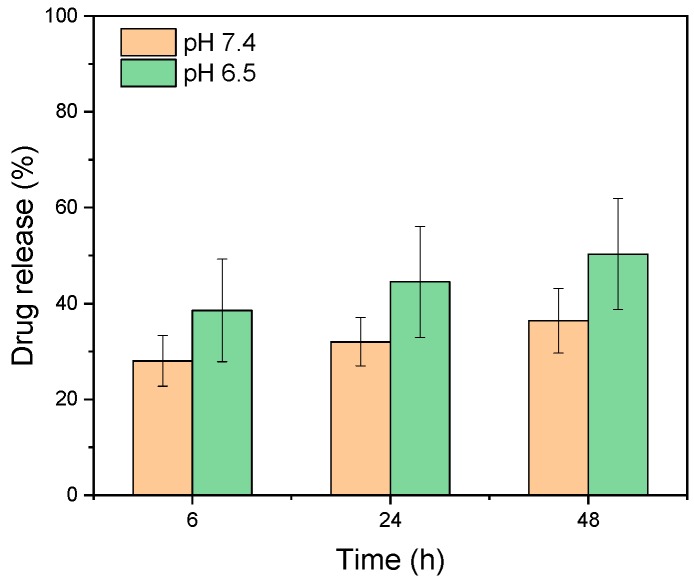
Drug leakage and release of IMQ-containing liposomes in different pH environments. IMQ: imiquimod.

Since there is slight change in the particle size and size distribution of liposomes in different pH environments, we have continued to investigate the drug leakage of liposomes in different pH environments in the hope of unraveling the effect of particle size changes on the drug stability in liposomes and the drug release behavior of nanocarriers in practice. According to the results (Figure 3), in either pH 7.4 or pH 6.5 environments, liposomes underwent burst release in the early stage. This result was consistent with the variation observed for the particle size. Particle-size changes led to structural changes in liposomes and caused drug release. Six hours later, the drug release slowed down. In addition, liposomes had a relatively greater drug release in a mildly acidic environment which could be due to the relatively more flexible lipid arrangement of DPPC in the acidic environment than in the neutral environment [17].

**Figure 4:**
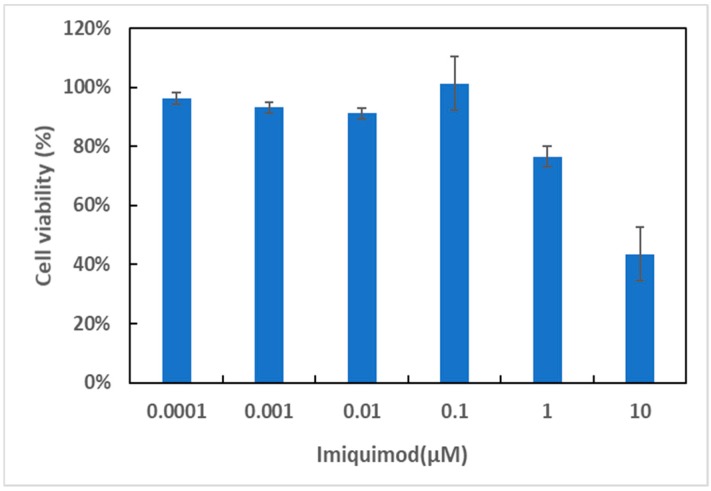
4T1 cytotoxicity at 24 h in the presence of IMQ-containing liposomes.

To understand the process of intracellular drug release of IMQ-containing liposomes, MTT was used to monitor the cytotoxicity of drug carriers in 4T1 cells in this study. Although imiquimod is used clinically as an immunotherapeutic drug for cancers [18], some articles have suggested that this drug also exhibits a mitochondria-dependent apoptosis pathway for cancer cells [19]. After the drug-containing liposomes were incubated with 4T1 cells for 24 h, the cancer-cell survival rate was only 44% at 10 µM (Figure 4), indicating that the drug was successfully released and caused cell death at a high concentration.

**Figure 5:**
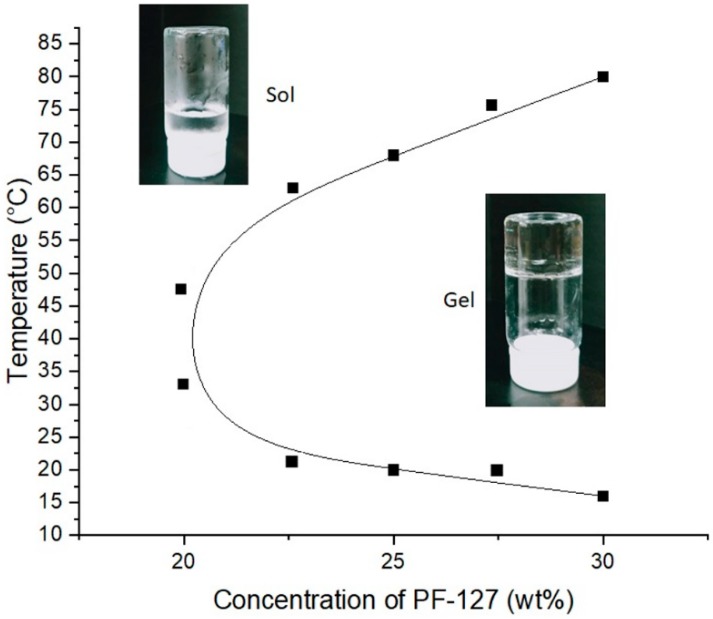
Phase diagram of PF127 at different concentrations and temperatures.

In order to achieve a sustained and slow release of drugs by the IMQ-containing liposomes at the locoregional position of the tumor, the drug carriers were mixed with the temperature-sensitive hydrogel PF127. PF127 is a thermal-reversible gel when in concentrations of 15%–30% w/w. It turns into gel when the temperature reaches body temperature (37 °C) and back into liquid at lower temperatures. This phenomenon is due to the fact that at low temperatures, PF127 is embedded in a layer of hydrates, while as the temperature increases, the hydrophilic segments of PF127 undergo desolvation, where the hydrogen bonds between solvent and chains are destroyed [20]. This phenomenon facilitates the interactions between hydrophobic segments of PF127, leading to gel formation. The sol–gel transition phase diagram of PF127 polymers is shown in Figure 5.

**Figure 6:**
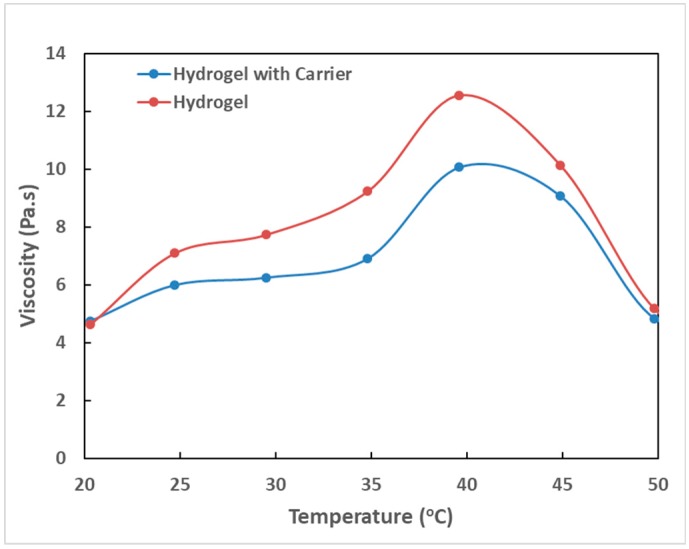
Analysis of the fluid viscosity of the hydrogel before and after mixing with IMQ-containing liposomes.

**Figure 7:**
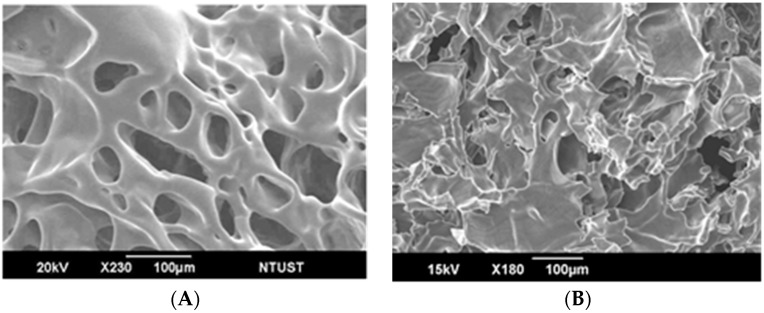
Examinations of the hydrogel combined with IMQ-containing liposomes by scanning electron microscopy: (**A**) pure PF127 and (**B**) PF127 mixed with IMQ-containing liposomes.

We next explored the gel formation process of the polymeric hydrogel and IMQ-containing liposome mixture in response to different temperatures. A rheometer was used to analyze the hydrogel embedded with IMQ-containing liposomes and it was found that the viscosity increased along with increasing temperature until approximately 40 °C, suggesting that the hydrogel retained its temperature-sensitive gel-forming property. At 40 °C, the hydrogel embedded with IMQ-containing liposomes had a viscosity slightly lower than the pure hydrogel. This was probably due to the fact that the added IMQ-containing liposomes reduced the hydrogen bond interactions between the temperature-sensitive polymers as shown in Figure 6. Further analysis of freeze-dried hydrogel samples by scanning electron microscopy revealed that the pure hydrogel contained hydrogel scaffolds with clearly formed pores. When the hydrogel mixed with IMQ-containing liposomes, the pores in the hydrogel became less obvious (Figure 7).

**Figure 8:**
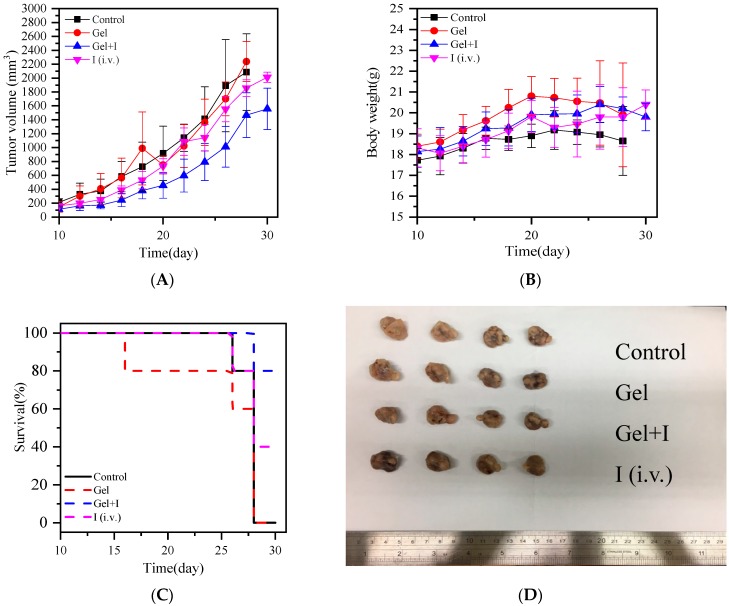
Breast cancer therapy assessment of liposome–hydrogel carriers: (**A**) tumor suppression ability analysis, (**B**) analysis of animal weight changes, (**C**) animal survival rate analysis, and (**D**) actual tumor sizes.

Finally, the therapeutic capacity of hydrogel mixed with IMQ-containing liposomes by in situ injection into breast cancer tissues was explored. The breast cancer model selected by the study was ductal carcinoma in situ. After the breast cancer tumor grew to a certain size, polymeric hydrogel and polymeric hydrogel mixed with IMQ-containing liposomes were injected in the tumor. IMQ-containing liposomes, administered by intravenous injection, served as the control group. The results, as shown in Figure 8A, suggest that the polymeric hydrogel alone did not possess the ability to suppress or promote tumor growth, and direct administration by intravenous injection could not effectively suppress tumor growth due to the lack of a high amount of drug accumulation in the tumor tissues. Compared to other test groups, the hydrogel with IMQ-containing liposomes displayed a higher suppression of tumor growth and the highest animal survival rate (Figure 8C). The polymeric hydrogel mixed with IMQ-containing liposomes also had no significant toxicity on the test mice, thus animal weight did not show a downward trajectory (Figure 8B). The body weight of mice was correlated to the healthy condition of tumor-bearing mice. A large body weight variation was found for the mice treated with only gel and the control group. For the tumor-bearing mice treated by the hydrogel loaded with IMQ-containing liposomes, the body weight variation of mice was within plus-minus 2 g. These results also indicate that the topical immune drug might be efficient in inhibiting the growth of tumors and keeping the healthy condition of the mice. In addition, by examining tumor tissues obtained after the test (Figure 8D), the growth of the breast cancer tumor was suppressed after the treatment of polymeric hydrogel mixed with IMQ-containing liposomes, with the tumor size being significantly smaller than other treatment methods. These results suggest that the hydrogel mixed with IMQ-containing liposomes developed in this study could be effectively used to treat breast cancer by delaying tumor growth and thus reducing the death rate.

## 4. Conclusions

By using the liposomes containing the immunotherapeutic anticancer drug imiquimod, this study explored the release of the drug in acidic and basic environments and analyzed the drug carrier (liposome) by mixing it with a temperature-sensitive hydrogel. At low temperatures, liposomes and temperature-sensitive polymers were in soluble forms; after the temperature increased to 37 °C, the liposome–temperature-sensitive-polymer water solution turned into a gel. By utilizing the temperature-sensitive gel-forming property, we injected this drug carrier in the proximity of the tumor tissues by in situ injection so as to achieve locoregional therapeutic specificity and reduced side effects on normal tissues. According to our findings, immunotherapeutic drug carriers have a better locoregional therapeutic effect than the traditional intravenous injection and thus present an alternative method for cancer therapy.

## References

[1] Witteveen A., Kwast A.B.G., Sonke G.S., Jzerman M.J.I., Siesling S. (2015). Survival after Locoregional Recurrence or Second Primary Breast cancer: Impact of the disease-free interval. PLoS ONE.

[2] Couzin-Frankel J. (2013). Breakthrough of the year 2013. Cancer immunotherapy. Science.

[3] Loi S., Michiels S., Salgado R., Sirtaine N., Jose V., Fumagalli D., Kellokumpu-Lehtinen P.L., Bono P., Kataja V., Desmedt C. (2014). Tumor Infiltrating Lymphocytes Are Prognostic in Triple Negative Breast Cancer and Predictive for Trastuzumab Benefit in Early Breast Cancer: Results from the FinHER Trial. Ann. Oncol..

[4] Hsu W.-H., Kao Y.-C., Chuang S.-H., Wang J.-S., Lai J.-Y., Tsai H.-C. (2019). Thermosensitive double network of zwitterionic polymers for controlled mechanical strength of hydrogels. RSC Adv..

[5] Guo D.D., Xu C.X., Quan J.S., Song C.K., Jin H., Kim D.D., Choi Y.J., Cho M.H., Cho C.S. (2009). Synergistic Anti-tumor Activity of Paclitaxel-incorporated Conjugated Linoleic Acid-coupled Poloxamer Thermosensitive Hydrogel in Vitro and in Vivo. Biomaterials.

[6] Darge H.F., Andrgie A.T., Tsai H.C., Lai J.Y. (2019). Polysaccharide and Polypeptide based Injectable Thermo-Sensitive Hydrogels for Local Biomedical Applications. Int. J. Biol. Macromol..

[7] Andrgie A.T., Mekuria S.L., Addisu K.D., Hailemeskel B.Z., Hsu W.H., Tsai H.C., Lai J.Y. (2019). Non-Anticoagulant Heparin Prodrug Loaded Biodegradable and Injectable Thermoresponsive Hydrogels for Enhanced Anti-Metastasis Therapy. Macromol. Biosci..

[8] Yamazaki N., Sugimoto T., Fukushima M., Teranishi R., Kotaka A., Shinde C., Kumei T., Sumida Y., Munekata Y., Maruyama K. (2017). Dual-stimuli responsive liposomes using pH- and temperature-sensitive polymers for controlled transdermal delivery. Polym. Chem..

[9] Reddy T.L., Garikapati K.R., Reddy S.G., Reddy B.V.S., Yadav J.S., Bhadra U., Bhadra M.P. (2016). Simultaneous delivery of Paclitaxel and Bcl-2 siRNA via pH-Sensitive liposomal nanocarrier for the synergistic treatment of melanoma. Sci. Rep..

[10] Vu-Quang H., Vinding M.S., Nielsen T., Ullisch M.G., Nielsen N.C., Nguyen D.-T., Kjems J. (2019). Pluronic F127-Folate Coated Super Paramagenic Iron Oxide Nanoparticles as Contrast Agent for Cancer Diagnosis in Magnetic Resonance Imaging. Polymers.

[11] Nguyen D.T., Dinh V.T., Dang L.H., Nguyen D.N., Gang B.L., Nguyen C.T., Nguyen T.B.T., Thu L.V., Tran N.Q. (2019). Dual Interactions of Amphiphilic Gelatin Copolymer and Nanocurcumin Improving the Delivery Efficiency of the Nanogels. Polymers.

[12] Pelegrino M.T., Lima B.d.A., Nascimento M.H.M.d., Lombello C.B., Brocchi M., Seabra A.B. (2019). Biocompatible and Antibacterial Nitric Oxide-Releasing Pluronic F-127/Chitosan Hydrogel for Topical Applications. Polymers.

[13] Cidade M.T., Ramos D.J., Santos J., Carrelo H., Calero N., Borges J.P. (2019). Injectable Hydrogels Based on Pluronic/Water Systems Filled with Alginate Microparticles for Biomedical Applications. Molecules.

[14] Yeh M.Y., Zhao J.-Y., Hsieh Y.-R., Lin J.-H., Chen F.-Y., Chakravarthy R.D., Chung P.-C., Lin H.-C., Hung S.-C. (2017). Reverse thermo-responsive hydrogels prepared from Pluronic F127 and gelatin composite materials. RSC Adv..

[15] Choi J.S., Yoo H.S. (2010). Pluronic/chitosan hydrogels containing epidermal growth factor with wound-adhesive and photo-crosslinkable properties. J. Biomed. Mater. Res. Part A.

[16] Khattak S.F., Bhatia S.R., Roberts S.C. (2005). Pluronic F127 as a Cell Encapsulation Material: Utilization of Membrane-Stabilizing Agents. Tissue Eng..

[17] Gong K., Feng S.S., Go M.L., Soew P.H. (2002). Effects of pH on the stability and compressibility of DPPC/cholesterol monolayers at the air-water interface. Colloid. Surf. A.

[18] Chi H., Li C., Zhao F.S., Ng T.B., Jin G., Sha O. (2017). Anti-tumor activity of toll-like receptor 7 agnoist. Front. Pharmacol..

[19] Ahn M.Y., Kwon S.M., Cheong H.H., Park J.H., Lee J., Min S.K., Ahn S.G., Yoon J.H. (2012). Toll-like receptor 7 agonist, imiquimod, inhibits oral squamous carcinoma cells through apoptosis and necrosis. J. Oral Pathol. Med..

[20] Akash M.S.H., Rehman K. (2015). Recent progress in biomedical applications of Pluronic (PF127): Pharmaceutical perspectives. J. Control. Release.

